# Assessing Bacteriological Profiles and Targeting Resistant Bacterial Superbugs to Develop an Antibiogram Aiding in Antibiotic Selection

**DOI:** 10.7759/cureus.75574

**Published:** 2024-12-12

**Authors:** Bhargavi M, Karthikeyan R, Kumar B

**Affiliations:** 1 Department of Pharmacy Practice, School of Pharmacy, Sri Balaji Vidyapeeth, Puducherry, IND; 2 Department of Pharmacy Practice, Ratnam Institute of Pharmacy, Nellore, IND

**Keywords:** antibiogram, anti-microbial resistance, e.coli, mdr bacteria infection, ssi, superbugs

## Abstract

Introduction

The success of surgical procedures is becoming more threatened by the advent of multi-drug resistant (MDR) bacterial strains, sometimes known as superbugs. These resistant microorganisms frequently cause post-surgical infections, which raise morbidity, death, and medical expenses. With an emphasis on resistant strains, this seeks to create an antibiogram and a thorough microbiological profile of surgical infections in order to help choose the most effective antimicrobial therapy. The outcomes will lessen the effects of resistant microbial superbugs in surgical settings, optimize the use of antibiotics in turn, and improve infection control techniques.

Objectives

To identify and isolate the bacterial microbes that trigger post-surgical infections in patients. To determine the antibiotic resistance profiles of these bacterial isolates. To construct a local antibiogram for post-surgical infections, aiding in the appropriate selection of antibiotics.

Method

A prospective cross-sectional study was carried out in Government General Hospital, Nellore. Based on the sample size, 738 patients were taken. Patients were recruited based on eligibility criteria. A questionnaire was prepared, and details were collected. Gram-staining techniques were used to identify organisms. Biochemical tests were done to confirm the bacteria. Culture sensitivity tests were carried out by disk diffusion method to know the zone of inhibition. A structured antibiogram was developed.

Results

Of the 738 patients, 324 were found to have Surgical Site Infections (SSI). One-hundredand seventy two females were prone to SSI, which is a high number. Three-hudred and thirty eight organisms were identified among 324 SSI patients, mostly comprised of staphylococcus (28.6%) followed by *Escherichia coli* (*E.coli*) (21.3%). The gynecology department comprises more SSI followed by surgical (31.7%). Cephalosporins are commonly used antibiotics before and after surgery. It is found to be resistant to most organisms, whereas Gentamicin is found to be sensitive.

Discussion

It was evident that the more contaminated the wounds being operated on, the higher the SSI. Compared to scheduled elective surgeries, the infection rate for emergency procedures was nearly twice as high. There was no bacterial growth in 404 patients out of 738.

Conclusion

This study emphasizes the increasing difficulty in treating surgical infections caused by antibiotic-resistant organisms and the urgent need for accurate, data-driven methods of infection management. It is advised to employ an antibiogram to reduce the spread of resistant organisms because a significant percentage of samples are resistant to the most commonly used medications. National surveillance of microbes resistant to antibiotics must be established.

## Introduction

Surgical site infections (SSIs) represent one of the most common complications following surgery, contributing to significant morbidity, prolonged hospital stays, increased healthcare costs, and in some cases, mortality. The increasing prevalence of antimicrobial resistance (AMR) in surgical pathogens has exacerbated the challenges in managing these infections, making it crucial to accurately identify the responsible microorganisms and determine their antibiotic susceptibility profiles. Surgical infections, which occur as a result of bacterial contamination during or after surgery, are a leading cause of morbidity, extended hospital stays, and mortality. In recent years, multi-drug-resistant (MDR) and extensively drug-resistant (XDR) bacteria have emerged, collectively known as superbugs [1,2]. Despite big advances in antimicrobial therapies and infection strategies, antibiotic resistance represents an emergency situation, especially in those immunocompromised hosts. Specifically, infections occurring due to multidrug-resistant and gram-negative pathogens are more responsible for increased mortality rates and may leave few effective antimicrobial options [3].

Global burden of surgical infections and antimicrobial resistance: Surgical infections are a major cause of post-operative complications, with approximately 5-10% of all surgical patients developing some form of SSIs per Boucher et al. study [4]. These infections are associated with increased morbidity, the need for additional treatments (e.g., extended hospital stays, surgeries, or additional antimicrobial therapies), and higher healthcare costs. In recent years, the global burden of surgical infections has been complicated by the emergence of multidrug-resistant (MDR) and extensively drug-resistant (XDR) bacteria, which are increasingly found in both hospital and community settings, according to the World Health Organization(WHO) [5]. This growing resistance has led to limited therapeutic options, often resulting in the use of last-line antibiotics, such as carbapenems, which in turn, accelerates the development of even more resistant pathogens [6].

As per Bassetti and Righi, the rise of antimicrobial resistance in surgical pathogens is particularly concerning because SSIs are often treated with empiric broad-spectrum antibiotics like cephalosporins [3]. The need for surveillance systems that provide precise, localized data on pathogen profiles and their resistance patterns is now more critical than ever, as inappropriate use of antibiotics can further fuel resistance development [7].

Role of microbiological profiling and antibiograms: The identification of microorganisms involved in surgical infections and the determination of their resistance patterns is essential for appropriate treatment decisions. Microbiological profiling involves isolating and identifying the pathogens responsible for infections, while antibiograms provide a comprehensive overview of the susceptibility of these pathogens to a variety of antibiotics, according to the Daniel et al. study [8]. Together, these tools allow clinicians to tailor antibiotic therapy based on local resistance patterns rather than relying on broad-spectrum agents that may not be effective against resistant strains.

The creation of a local antibiogram is especially important, as resistance patterns can vary significantly from one healthcare setting to another. Studies have shown that resistance profiles can differ based on geographic region, the type of surgery performed, patient population, and hospital-level factors such as infection control measures and antimicrobial stewardship programs [4,8]. By collecting and analyzing data from local microbiological surveillance, hospitals can develop antibiograms that reflect the pathogens most frequently encountered in their specific surgical population, ensuring that empiric antibiotic therapies are aligned with the most common and most resistant organisms in that setting.

Common pathogens in surgical infections and their resistance profiles: The pathogens most frequently associated with surgical infections include *Staphylococcus aureus*, *Escherichia coli *(*E.coli*), *Klebsiella pneumoniae *(*K. pneumoniae*), and *Pseudomonas aeruginosa*, among others. *Staphylococcus aureus*, including methicillin-resistant strains (MRSA), is a leading cause of wound infections, particularly following orthopedic and cardiovascular surgeries [9]. Additionally, Enterobacteriaceae, such as *E. coli* and *K. pneumoniae*, are common culprits in gastrointestinal and abdominal surgeries. These pathogens have increasingly become resistant to commonly used antibiotics, including beta-lactams, fluoroquinolones, and aminoglycosides, leading to treatment failures in surgical patients [8].

One of the most concerning developments in the realm of surgical infections is the emergence of carbapenem-resistant Enterobacteriaceae (CRE), which produce enzymes that inactivate beta-lactam antibiotics, including the carbapenems that are often used as last-resort treatments [10]. These resistant organisms pose significant challenges in both treatment and infection control, as they often spread easily within hospital environments, complicating surgical care and leading to more severe patient outcomes [5].

Antimicrobial stewardship and infection control: Effective management of SSIs requires not only timely and accurate diagnosis of the infecting pathogens but also adherence to robust infection control practices and antimicrobial stewardship programs [7]. Studies have demonstrated that incorporating antimicrobial stewardship into clinical practice reduces the incidence of resistant infections and improves patient outcomes [4]. By developing targeted therapies based on the results of local microbiological profiling and antibiograms, hospitals can significantly reduce the use of broad-spectrum antibiotics, which are often overprescribed in the absence of culture data [8].

This study aims to address this issue by developing a comprehensive microbiological profile and antibiogram targeting resistant bacterial pathogens in surgical infections. The ultimate goal of this research is to provide healthcare professionals with a powerful tool to inform clinical decisions and improve outcomes for patients undergoing surgery. By understanding the resistance patterns prevalent in specific surgical settings, we can enhance infection prevention strategies, optimize antibiotic stewardship, and reduce the impact of antibiotic-resistant superbugs in surgical infections.

## Materials and methods

The methodology includes study design, participant selection, sample collection, laboratory analysis techniques, antibiotic susceptibility testing, data analysis procedures, and ethical considerations.

Study design

This study was a prospective, observational cross-sectional study conducted at Government General Hospital, Nellore, a tertiary care facility that performs a wide variety of surgical procedures. The study was approved by the Institutional Review Board (IRB) at Ratnam Institute of Pharmacy with approval number Ratnam/Pharmacy/Res/DPP/IRB/2019-02. The research was designed to evaluate the microbiological characteristics and antibiotic resistance patterns of bacterial pathogens isolated from post-surgical infections. The study aimed to provide localized data on pathogen prevalence and resistance profiles to guide clinicians in the selection of appropriate antibiotics for treating surgical infections. The informed consent was obtained from all patients involved in the research. Data collection, analysis, and reporting were done in accordance with ethical guidelines for research involving human subjects.

Participant selection

The study included patients who developed surgical site infections (SSIs) following elective or emergency surgeries performed at the hospital. 

Inclusion Criteria

Patients aged 18 years and older, patients who underwent any type of surgery, including abdominal, orthopedic, cardiovascular, and soft tissue surgeries, and patients who exhibited clinical signs of a surgical site infection (e.g., redness, swelling, pain, purulent discharge) within 30 days post-surgery willing to participate were included in the study.

Exclusion Criteria 

Patients with pre-existing infections at the time of surgery, patients who were diagnosed with infections outside the surgical site (e.g., urinary tract infections, respiratory infections), and patients who refused to participate in the study were excluded.

Sampling procedure

A questionnaire was used to obtain data from the patient after obtaining an informed consent from the patient. A total of 738 patients were enrolled in the study. For each patient, clinical data, including age, sex, surgical procedure, and co-morbid conditions were recorded.

Sample collection

The primary samples for microbiological analysis were obtained from surgical wounds showing signs of infection. Samples were collected following standard aseptic techniques to avoid contamination. Wound swabs, pus swabs, blood cultures, urine cultures etc were collected. Samples were collected within 48 hours of detecting signs of infection to ensure the most accurate representation of the pathogen causing the infection.

Microbiological analysis

Upon arrival in the laboratory, samples were processed using standard microbiological techniques to isolate and identify the bacterial pathogens. The microbiological analysis involved the following steps. (1) Gram staining* - a*ll samples were subjected to Gram staining methods like primary stain & Iodine solution to provide an initial differentiation of Gram-positive and Gram-negative organisms; ​​​​​​​(2) Bacterial culture - each sample was inoculated onto appropriate selective and differential media, including (a) MacConkey agar for the isolation of Gram-negative bacteria, particularly Enterobacteriaceae and non-fermenters, (b) Blood agar for the growth of Gram-positive cocci (e.g., *Staphylococcus aureus*, Streptococcus species), and (c) Eosin Methylene Blue (EMB) agar for the isolation of *Escherichia coli* and coliforms; (3) ​​​​​​​Incubation - plates were incubated at 37°C for 18-24 hours, and growth was monitored at regular intervals. Cultures showing growth were subcultured onto fresh media to ensure purity and obtain isolated colonies. (3) Identification of pathogens - identification of bacterial species was done using standard biochemical tests like the catalase test; (4) ​​​​​​​Antibiotic resistance testing - after pathogen identification, antibiotic susceptibility testing was conducted using the Kirby-Bauer method; (5) Construction of local antibiogram - the antibiotic susceptibility data from all bacterial isolates were compiled to construct a local antibiogram, which provides a detailed summary of the resistance patterns of bacterial pathogens identified from post-surgical infections. The antibiogram categorized each bacterial species according to its resistance to key antibiotics commonly used in surgical settings.

For each pathogen, the percentage of isolates resistant, intermediate, or susceptible to each antibiotic was calculated. The data was stratified by bacterial species and type of surgery to assess any patterns related to specific surgical interventions. The antibiogram was then presented as a graphical representation to aid clinicians in selecting empiric antibiotic therapy based on the most prevalent resistant pathogens in the institution.

Data analysis

Descriptive statistics were used to analyze the data, including the frequency of bacterial isolates, antibiotic resistance patterns, and the distribution of resistance by surgical procedure. The Chi-square test was applied to evaluate associations between patient demographics and types of surgery. The confidence intervals (CIs) and p-values were calculated to assess statistical significance, with a p-value <0.05 considered significant. The data was analyzed using SPSS statistical software, version 20 (IBM Corp., Armonk, NY), to determine the most common bacterial pathogens and the distribution of resistance patterns and to identify any emerging trends in antimicrobial resistance.

## Results

Patients' profiles

**Figure 1:**
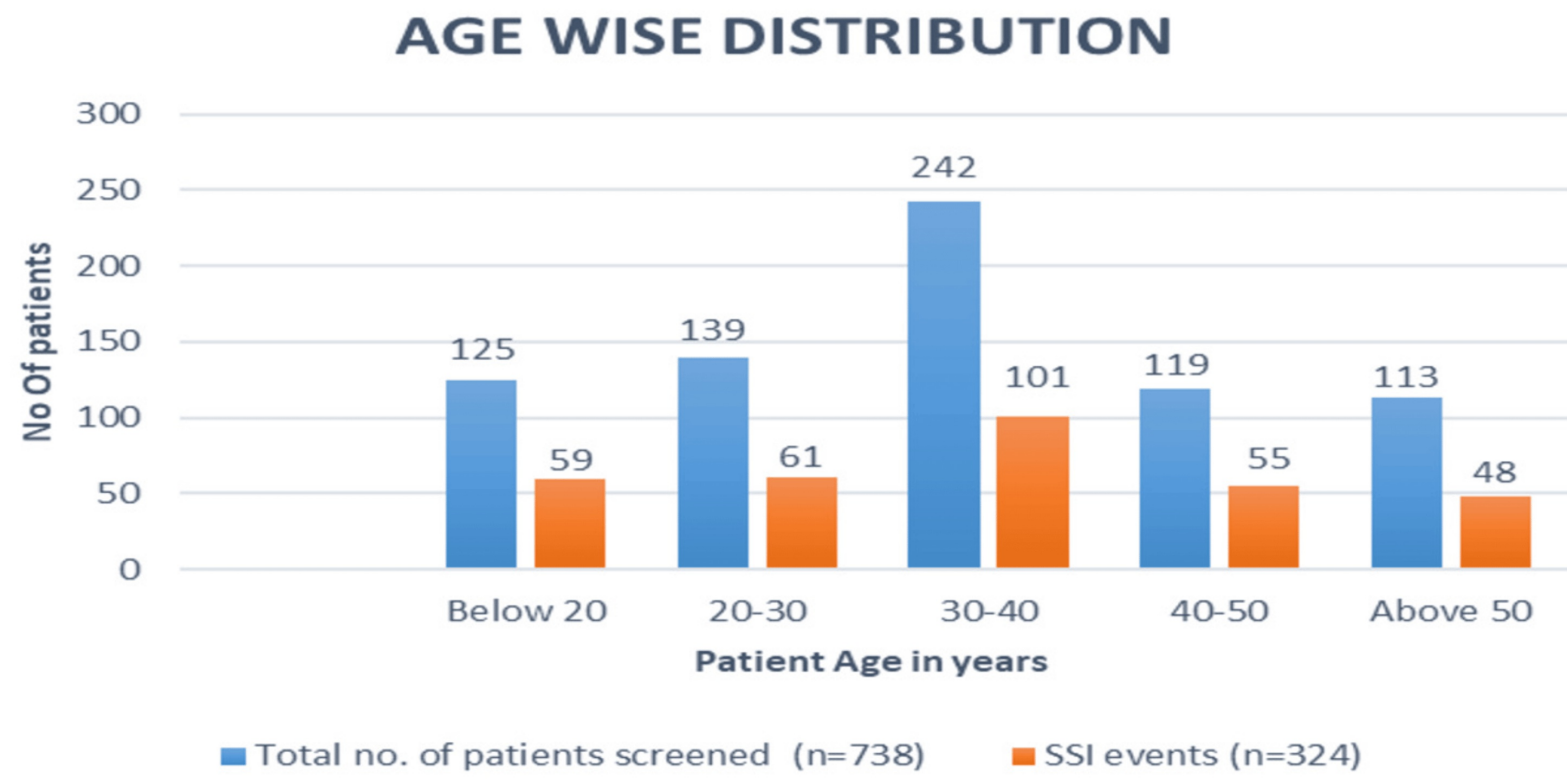
Age wise distribution

A total of 738 patient details were collected, and out of those, 324 patients were found to be affected with surgical site infections (SSI). The overall incidence rate was 43.9%. Out of 738 patients, the most affected age group was 30-40 years of age, with an SSI rate of 31.1% (Figure 1).

Out of 738, 413 were males and 325 were females. Table 1 shows a detailed description of the total patients screened with SSI events based on different variables. As per the total SSI, females are more affected. Regarding the departments, the rate was high for gynecology, whereas the ENT department showed a greater rate of 62.2%, followed by gynecology at 58.6%. Emergency surgeries showed higher SSI than elective. More SSI was seen in clean, contaminated wounds.

**Table 1:** Association of SSI events with different variable characteristics SSI-Surgical site infections; ENT-Ear, Nose and Throat.

Variable	Total no. of patients screened (n=738)	No SSI events, n=414 (56%)	SSI events, n =324 (44%)
Patient gender			
Female	413	241 (58.3%)	172 (41.7%)
Male	325	173 (53.2%)	152 (46.8%)
Hospital stays			
Below 10 days	234	110 (47%)	124 (53%)
Above 10 days	504	304 (60.3%)	200 (39.7%)
Department			
General surgery	323	221 (68.4%)	102 (31.6%)
Gynecology	181	75 (41.4%)	106 (58.6%)
Orthopedics	104	56 (53.8%)	48 (46.1%)
Ophthalmology	69	39 (56.5%)	30 (43.5%)
ENT	61	23 (37.7%)	38 62.2%)
Type of surgery			
Elective	658	392 (59.6%)	266 (40.4%)
Emergency	80	22 (27.5%)	58 (72.5%)
Type of surgery			
Clean wound	252	127 (50.3%)	125 (49.7%)
Clean contaminated wound	365	193 (52.9%)	172 (47.1%)
Contaminated wound	119	92 (77.3%)	27 (22.7%)
Dirty wound	2	2 (100%)	0 (0%)

SSI events

**Figure 2:**
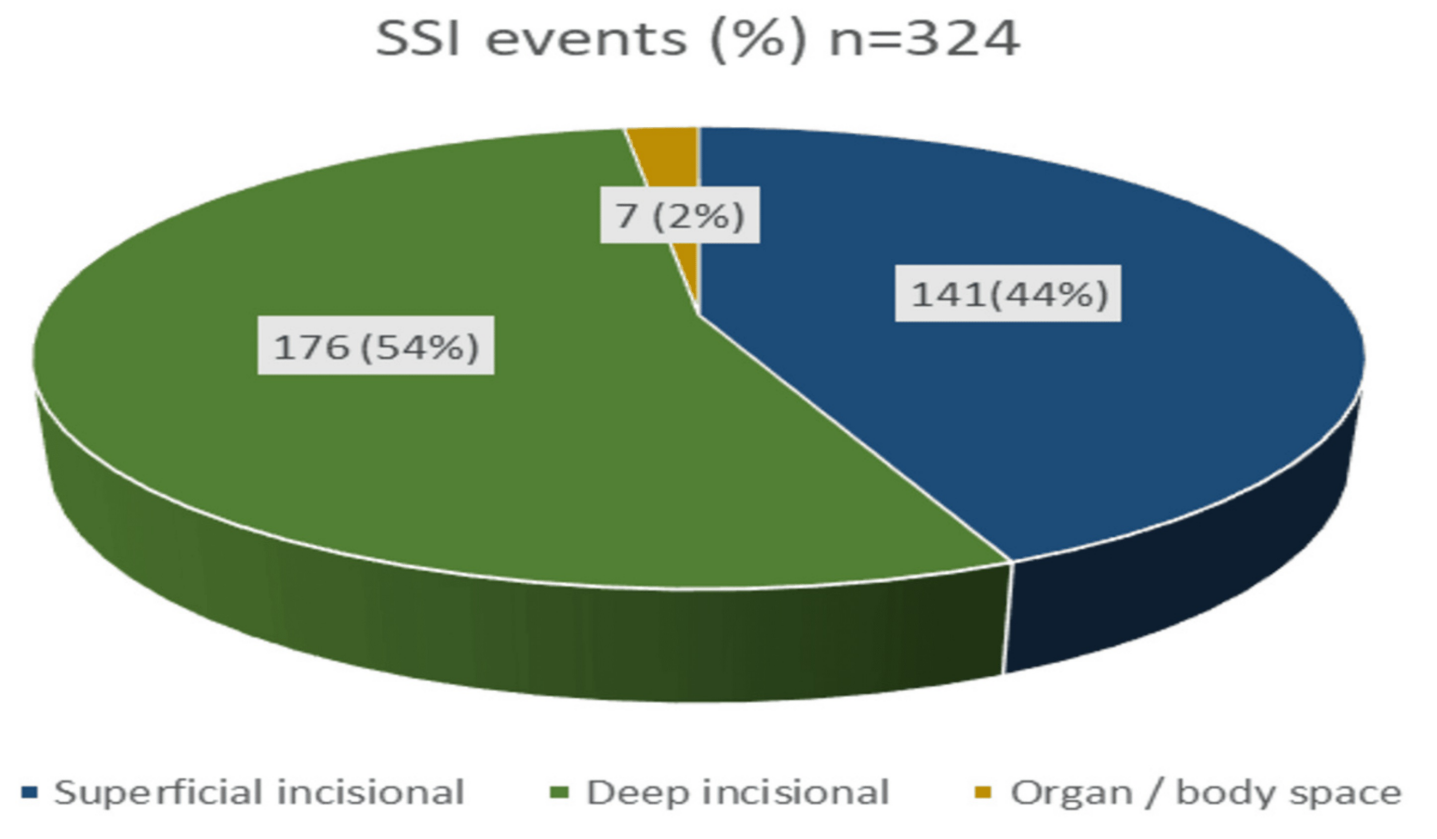
Distribution based on type of SSI

Among 324 SSI events, deep incisionals were higher, followed by superficial incisionals. The most common clinical manifestations observed were redness, warmth, fever, and delayed healing. SSIs were mostly seen in patients after more than five days of surgery, followed by the third day of surgery (Figure 2).

Prescribing pattern

**Figure 3:**
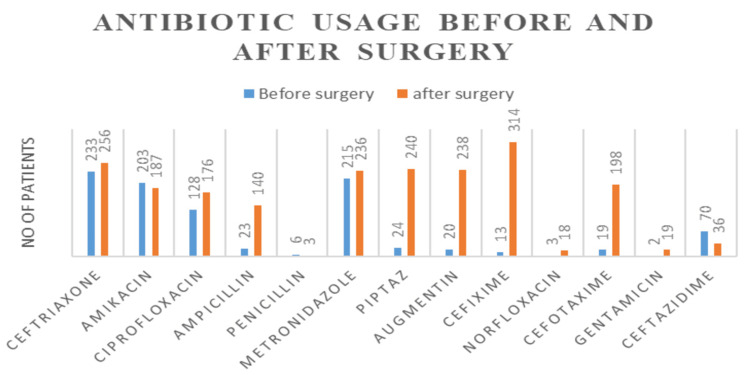
Antibiotic usage before and after surgery

A group of antibiotics were used before and after surgeries. Among 738 patients, 172 were not given any antibiotics before surgery, of which 91 were affected with SSI. Of the 738 patients, 142 were not given any antibiotics after surgery, of which 99 were affected with SSI. Ceftriaxone was used most commonly before surgery followed by metronidazole. Cefixime was used most commonly after surgery followed by ceftriaxone (Figure 3).

Among all the surgical procedures done the SSI were found mostly in hysterectomy (81.8%) followed by tubectomy surgery (70.3%). The remaining surgeries are explained in Table 2.

**Table 2:** Incidence of surgical site infections in relation to different types of surgical procedures SICS+PCIOL-Small incision cataract surgery with posterior chamber intraocular lens.

Type of surgical procedures	No. of patients (738)	Frequency and percentage of surgical site infections (%)
Appendicectomy	131	46 (35.1%)
Hydrocelectomy	36	10 (27.8%)
Hysterectomy	22	18 (81.8%)
Surgical debridement	45	23 (51.1%)
Herniotomy	59	15 (25.4%)
Tubectomy	101	71 (70.3%)
Mastectomy	20	2 (10%)
Tendon repair	58	30 (51.7%)
Knee replacement	46	18 (39.1%)
SICS + PCIOL	69	30 (43.5%)
Caesarian	58	17 (29.3%)
Tonsillectomy	38	22(57.9%)
Tympanoplasty	23	16(69.6%)
Others	32	6(18.7%)

Bacteriological findings

Three-hundred and thirty-eight organisms were identified from 324 SSI. The type of specimen includes blood, pus, urine, fluid, ear swabs, and corneal scraping. As per Table 3, Staphylococcus was the most found organism, followed by *E.coli*, Pseudomonas, Streptococcus, and Gonococci, which were the least markable. The department-wise distribution of organisms is shown in Table 4.

**Table 3:** Distribution of organisms isolated from various clinical samples *E.coli* - *Escherichia coli*

Specimen type	Staphylococcus	Streptococcus	E. coli	Klebsiella	Pseudomonas	Gonococci	Pneumococci	Proteus
Blood	42	14	36	12	12	4	4	6
Pus	12	12	10	6	4	2	4	-
Fluid	9	2	2	4	8	-	2	3
Urine	24	13	24	11	3	2	-	4
Ear swab	2	-	-	-	11	-	-	4
Corneal scrapping	8	5	-	-	12	2	3	-
Total	97	46	72	33	50	10	13	17
(%)	28.6%	13.6%	21.3%	9.7%	14.7%	2.9%	3.8%	5.02%

**Table 4:** Bacterial distribution of microorganisms from various departments ENT- Ear, Nose and Throat; *E.coli* - *Escherichia coli.*

Microorganism Isolated	Departments				
	Surgical	Gynecology	Orthopedics	Ophthalmology	ENT
Pseudomonas	7	0	14	12	17
Klebsiella	6	23	4	0	0
E. Coli	16	54	2	0	0
Proteus	3	1	3	0	10
Pneumococci	2	6	2	3	0
Gonococci	8	0	0	2	0
Staphylococcus	38	22	15	8	14
Streptococcus	27	3	11	5	0
Total(n=338)	107	109	51	30	41
(%)	31.7%	32.2%	15.08%	8.8%	12.1%

Culture results

The majority of culture specimens have bacterial growth within 48 hours of incubation. The majority of them were sensitive to gentamycin and resistance to ceftriaxone. Most of the staphylococcus organisms were resistant to metronidazole and sensitive to gentamycin and ceftazidime. Most of the *E.coli *organisms were resistant to ampicillin and sensitive to gentamycin. The sensitivity pattern is shown in Table 5.

**Table 5:** Antibiogram *E.coli* - *Escherichia coli.*

Organism	Antimicrobial Agents													
	Pattern	Ceftriaxone	Amikacin	Gentamicin	Ampicillin	Ciprofloxacin	Ceftazidime	Penicillin	Metronidazole	Piptaz	Augmentin	Cefixime	Norfloxacin	Cefotaxime
Staphylococcus n= 97	R S	82 15	NT	07 90	NT	59 38	17 80	69 28	71 26	20 77	NT	60 37	NT	56 41
Streptococcus n= 46	R S	24 22	31 15	04 42	NT	29 17	15 31	12 34	NT	33 16	23 23	32 14	NT	NT
E. coli n= 72	R S	58 14	46 26	08 64	66 06	55 17	24 48	55 17	NT	28 44	NT	NT	26 44	27 45
Klebsiella n= 33	R S	14 19	11 22	11 22	13 20	10 23	08 25	NT	NT	NT	13 20	NT	12 21	NT
Pseudomonas n= 50	R S	26 24	27 23	13 37	NT	27 23	22 28	32 18	NT	NT	20 30	19 31	NT	32 18
Pneumococci n= 13	R S	08 05	09 04	NT	04 09	00 13	NT	08 05	NT	NT	NT	NT	4 9	NT
Gonococci n= 10	R S	03 07	06 04	NT	06 04	03 07	NT	NT	NT	4 6	NT	NT	2 8	NT
Proteus n= 17	R S	08 09	17 00	00 17	10 07	NT	NT	NT	NT	13 4	NT	NT	12 5	NT

## Discussion

The use of antibiotics in surgical patients both for the prophylaxis and treatment of infections is a justifiable practice that, however, requires a regular review of the chosen regimen on the grounds of efficacy, toxicity, hospital stay, and other aspects to maximize the benefits to the patient. The present study attempts to assess the general pattern of how antibiotics are used in surgical wards rather than attempting to judge individual prescriptions as appropriate or inappropriate. The present study is about to know the present status of antibiotic resistance patterns of common bacterial isolates in a tertiary care hospital of Nellore region, Andhra Pradesh. The overall SSI rate determined in this study was 43.9% out of 324 patients.

Based on age-wise categorization, 30-40 years age group patients are more prone to SSI than other age groups. As per statistical analysis, there was no strong clinical association between SSIs and age group (P > 0.05, by using the Chi-square test). Our study was supported by the Seni et al. study [11]. The incidence of SSI was more common in males (46.8%) than in females (41.7%). In their study, Mama et al. [12] stated that males were more prone than females, which supports our study findings.

In our study, the infection rate is higher in patients who stayed below 10 days in hospital than the patients who stayed above 10 days. As per statistical analysis, it is found that there is no strong clinical association between SSIs and hospital stay (P > 0.05, by using the Chi-square test).

We have recruited patients from General Surgery, Gynecology, Orthopedics, Ophthalmology, and ENT departments in which ENT (62.2% of SSI’s from total ENT cases) had shown more risk to SSI followed Gynecology department (58.6% of SSI’s from total Gynecology cases). There was a strong clinical association between SSIs and department as per statistical analysis (P < 0.05, by using the Chi-square test).

It was evident that SSI increases with an increase in the degree of contamination of the wounds operated upon. The infection rate was found to be almost two times higher in emergency procedures than in planned elective procedures. As per statistical analysis, there was a strong clinical association between SSIs and type of surgery (P < 0.05, by using the Chi-square test; odds ratio (OR) = 0.08). In a study conducted by Koul et al., the infection rate encountered in emergency surgeries was triple the rate of elective surgeries [13]. Several other studies also corroborate the evidence that emergency surgeries are more prone to wound infections. According to the study, the number of SSIs is influenced by the duration of surgery. The surgical patients with a duration between 45 mins to 1 hr (47.54%) were found to be more prone to SSI. As per statistical analysis, there was a strong clinical association between SSIs and the duration of surgery (P < 0.05, by using the Chi-square test).

Based on the type of SSI, the majority of infections were deep incisional than superficial incisional and organ/body space infections. As per statistical analysis, there was a strong clinical association between SSIs and the type of wound (P < 0.05, by using the Chi-square test). Our study revealed that the patients who received antibiotic prophylaxis (30.41%) were more prone to infection. Ceftriaxone, a third-generation cephalosporin mostly used before surgeries, has shown resistance to most of the organisms. Among post-operative patients who did not receive antibiotics, 42.30% developed more infections.

According to our study, hysterectomy (81.8%) has more SSI followed by tubectomy (70.3%), tympanoplasty (69.6%), tendon repair (51.7%), surgical debridement (51.1%), small incision cataract surgery with posterior chamber intraocular lens (SICS+PCIOL) (43.5%), knee replacement (39.1%), appendicectomy (35.1%), herniotomy (25.4%), mastectomy (10%), hydrocelectomy (27.8%) had shown infection.

Four hundred and four patients out of 738 had no bacterial growth. This may be the result of the body's defense mechanism overpowering the bacteria during the normal healing process and antimicrobial activity in the bloodstream because all of the patients were receiving antibiotic therapy after surgery. Additionally, since cultures were incubated aerobically, it's probable that some organisms were anaerobic bacteria that were overlooked. As a result, such creatures could not grow in this environment.

In our investigation, among 324 SSI patients, 338 organisms were isolated. On performing the culture sensitivity test, the most common bacteria isolated was Staphylococcus (28.6%) followed by *E.coli* (21.3%), Pseudomonas (14.7%), Streptococcus (13.6%), Klebsiella (9.7%), Proteus (5.02%), Pneumococci (3.8%) and Gonococci (2.9%). Our study was supported by various studies stating Staphylococcus was the most commonly isolated pathogen in their studies. The findings also agree with those of Anguzu and Olila's study which identified *Staphylococcus aureus* as the commonest causative agent of potentially contaminated wounds [14]. Because *Staphylococcus aureus* is an endogenous source of infection, it may be quite prevalent. Environmental contamination, such as that seen in surgical instruments, can potentially result in infection with this organism. *S. aureus*, a common surface bacteria, can easily enter surgical areas due to the disturbance of the normal skin barrier.

In our study, we found that in the surgical department, Staphylococcus was the most isolated and Pneumococci were the least isolated organisms; in the Gynecology department, *E.coli* was the most isolated bacterial pathogen; in the Orthopedics department, Staphylococcus was the most identified pathogen and Gonococci was the least identified in zero samples, in Ophthalmology and ENT departments, Pseudomonas was the most identified pathogen.

Our investigations found that the majority of Staphylococcus isolates were highly resistant to ceftriaxone and metronidazole and highly sensitive to gentamicin and ceftazidime. Streptococcus isolates were highly resistant to piperacillin/tazobactam (Piptaz) and highly sensitive to amikacin. *E.coli* isolates were highly resistant to ampicillin and highly sensitive to ceftazidime. Klebsiella isolates were highly resistant to ampicillin and highly sensitive to amikacin. Pseudomonas isolates were highly resistant to penicillin and highly sensitive to gentamicin and ceftazidime. Pneumococci isolates were highly resistant to ceftriaxone and highly sensitive to ciprofloxacin. Gonococci isolates were highly resistant to amikacin and ampicillin and highly sensitive to ceftriaxone and ciprofloxacin. Proteus isolates were highly resistant to ampicillin and highly sensitive to gentamicin. The majority of *Staphylococcus aureu*s were sensitive to gentamicin. This finding is similar to the study done in Mulago Hospital by Wewedru Izale C, where the sensitivity of *S. aureus *to gentamicin was 70% [15]. Ceftriaxone has become resistant in the recent past, as stated in the studies conducted in the US, and in our study too ceftriaxone showed resistance to most of the pathogens isolated. Gentamicin and ceftazidime are highly sensitive to most of the isolated bacterial pathogens. Susceptibility outcome revealed that gentamycin was the most effective antibiotic. Similar observations have been reported in the study conducted by Andhoga et al. [16].

Limitations

While this study provides valuable insights, there are limitations that should be addressed in future research. First, the study was conducted in a single healthcare facility, which may limit the generalizability of the findings to other settings with different patient populations or infection control practices. Future studies could expand to multicenter trials to obtain a more comprehensive view of regional or national resistance patterns.

Second, while the study focused on bacterial pathogens, the role of fungal infections, particularly in immunocompromised patients, was not explored. In some surgical contexts, fungi such as Candida species can also cause significant infections, and their inclusion in future studies would provide a more complete picture of post-surgical infections.

Finally, the evolving nature of antimicrobial resistance demands ongoing research into novel treatment strategies, including the development of new antibiotics, bacteriophage therapy, and alternative antimicrobial agents. Future studies should investigate the potential of these emerging therapies in managing surgical infections caused by resistant pathogens.

## Conclusions

This study underscores the growing challenge of antibiotic-resistant pathogens in surgical infections and highlights the critical need for precise, data-driven approaches to managing these infections. Our findings confirm the significant prevalence of multi-drug-resistant bacteria. These pathogens not only complicate treatment regimens but also contribute to prolonged hospital stays, increased mortality rates, and higher healthcare costs.

In conclusion, the integration of microbiological surveillance-targeted antibiograms and robust infection control protocols is vital for addressing the growing threat of resistant pathogens in surgical infections. As antimicrobial resistance continues to challenge the efficacy of conventional therapies, a comprehensive, localized approach to infection management will be essential to ensuring optimal clinical outcomes and safeguarding the effectiveness of antibiotics for future generations.

## Appendices

                       PATIENT DATA COLLECTION FORM/ QUESTIONNAIRE

Patient Name:                                                           Age/Gender:             

Admission No.:                                                         Department/Ward/Unit:

Date of Admission:                                                   Date of Surgery:      

Chief Complaints:

Past Medical History:

 (1) DM (2) Prolonged Steroid usage (3) Hypertension (4) Others (5) None

Past Medication History:

Family history:

Personal/Social history and habits:

Alcoholic/smoker:                                                      Exercise:

Diet (veg/non-veg):                                                    Bowel & Bladder habits:

Hospital stays in days: 1) Below 10 days 2)   Above 10 days

History of previous use of antibiotics within one month: Yes   /   No

If yes, what type of antibiotics:

For how long have been in such antibiotics:

Pre-operative diagnosis:

Type of wound: 1) Clean wound 2) Clean contaminated wound 3) Contaminated wound 4) Dirty wound

Type of surgery: a) Emergency surgery b) Elective surgery

Timing of surgical antimicrobial prophylaxis: 1) Before the operation 2) During operation 3) After operation 4) Not initiated at all.

Prophylactic antibiotics administered:

RX:

Surgical procedure performed:

Duration of operation in minutes: a) 0-45 b) 46-120 c)  >120

Any Surgical Site Infections observed: Yes/No

Clinical manifestations of SSI: 1) Redness 2) Warmth 3) Swelling 4) Pus discharge 5) Pain 6) Gaping at the operation site 7) Fever 8) Delayed healing 9) Others

Day of infection after surgery: a) 2nd b) 3rd c) 4-5days d) More than 5days

Type of infection: a) Superficial SSI b) Deep SSI c) Organ/Space SSI

Risk Factors of SSI: 1) DM 2) Existing infection 3) MRSA carrier 4) Old age 5) Obesity 6) Malnutrition 7) Hypothermia 8) Hypoxia 9) Hair removal 10) Prolonged preoperative stay 11) Duration of surgery 12) Multiple assistance 13) Surgical procedure 14) Tissue injury 15)  Intra-operative contamination.

Post-operative antibiotics RX:

Laboratory results

Culture swab: a) Blood b) Pus c) Fluid d) Urine e) Ear swab f) Corneal scrapping

Type of Organism isolated:

Sensitivity pattern of isolated organisms

Sensitivity to Antibiotics:

Resistance to Antibiotics:

## References

[1] Centers for Disease Control and Prevention (CDC). (2024). 2019 Antibiotic Resistance Threats Report. https://www.cdc.gov/antimicrobial-resistance/data-research/threats/index.html.

[2] (2022). Global burden of bacterial antimicrobial resistance in 2019: a systematic analysis. Lancet.

[3] Bassetti M, Righi E (2013). Multidrug-resistant bacteria: what is the threat?. Hematology Am Soc Hematol Educ Program.

[4] Boucher HW, Talbot GH, Bradley JS (2009). Bad bugs, no drugs: no ESKAPE! An update from the Infectious Diseases Society of America. Clin Infect Dis.

[5] (2024). Antimicrobial resistance. https://www.who.int/news-room/fact-sheets/detail/antimicrobial-resistance.

[6] Magiorakos AP, Srinivasan A, Carey RB (2012). Multidrug-resistant, extensively drug-resistant and pandrug-resistant bacteria: an international expert proposal for interim standard definitions for acquired resistance. Clin Microbiol Infect.

[7] Barie PS (2013). Guidelines for antimicrobial prophylaxis in surgery: a must-read, must-heed for every surgeon. Surg Infect (Larchmt).

[8] Raymond DP, Kuehnert MJ, Sawyer RG (2002). Preventing antimicrobial-resistant bacterial infections in surgical patients. Surg Infect (Larchmt).

[9] Paterson DL (2006). Resistance in gram-negative bacteria: enterobacteriaceae. Am J Med.

[10] Ma J, Song X, Li M (2023). Global spread of carbapenem-resistant Enterobacteriaceae: epidemiological features, resistance mechanisms, detection and therapy. Microbiol Res.

[11] Seni J, Najjuka CF, Kateete DP (2013). Antimicrobial resistance in hospitalized surgical patients: a silently emerging public health concern in Uganda. BMC Res Notes.

[12] Mama M, Abdissa A, Sewunet T (2014). Antimicrobial susceptibility pattern of bacterial isolates from wound infection and their sensitivity to alternative topical agents at Jimma University Specialized Hospital, South-West Ethiopia. Ann Clin Microbiol Antimicrob.

[13] Koul N, Kakati B, Agarwal S, Mittal G (2022). Multidrug resistant Acinetobacter baumannii causing Ventilator associated respiratory infections at a tertiary care center of India. IP Int J Med Microbiol Trop Dis.

[14] Anguzu JR, Olila D (2007). Drug sensitivity patterns of bacterial isolates from septic post-operative wounds in a regional referral hospital in Uganda. Afr Health Sci.

[15] Wewedru Izale C (2002). Epidemiology of antimicrobial resistance in pathogens isolated in Mulago Hospital in 1998 and 2000. African Index Medicus (AIM).

[16] Andhoga J, Macharia AG, Maikuma IR, Wanyonyi ZS, Ayumba BR, Kakai R (2002). Aerobic pathogenic bacteria in post-operative wounds at Moi Teaching and Referral Hospital. East Afr Med J.

